# System Efficiency of a Tap Transformer Based Grid Connection Topology Applied on a Direct Driven Generator for Wind Power

**DOI:** 10.1155/2014/784295

**Published:** 2014-08-31

**Authors:** Senad Apelfröjd, Sandra Eriksson

**Affiliations:** Swedish Center for Renewable Electric Energy Conversion, Division for Electricity, Uppsala University, Box 534, 751 21 Uppsala, Sweden

## Abstract

Results from experiments on a tap transformer based grid connection system for a variable speed vertical axis wind turbine are presented. The tap transformer based system topology consists of a passive diode rectifier, DC-link, IGBT inverter, LCL-filter, and tap transformer. Full range variable speed operation is enabled by using the different step-up ratios of a tap transformer. Simulations using MATLAB/Simulink have been performed in order to study the behavior of the system. A full experimental set up of the system has been used in the laboratory study, where a clone of the on-site generator was driven by an induction motor and the system was connected to a resistive load to better evaluate the performance. Furthermore, the system is run and evaluated for realistic wind speeds and variable speed operation. For a more complete picture of the system performance, a case study using real site Weibull parameters is done, comparing different tap selection options. The results show high system efficiency at nominal power and an increase in overall power output for full tap operation in comparison with the base case, a standard transformer. In addition, the loss distribution at different wind speeds is shown, which highlights the dominant losses at low and high wind speeds. Finally, means for further increasing the overall system efficiency are proposed.

## 1. Introduction

The number of wind turbines connected to the electrical grid is increasing fast [1]. Today's commercially available wind turbines combine a variety of innovative concepts with proven technologies. Among the systems there are a few clear groups of topologies and solutions. The first system characteristic that can be identified is whether the turbine is designed to operate at fixed or variable speed. This system characteristic gives a clear division of concepts into two main areas, where we in this work address a full variable speed topology. Fixed speed turbines were the dominating topologies in the early 1990s where the standard installed wind turbine was of the fixed speed type. During the last decade variable speed wind turbines have become the dominating type among the installed wind turbines around the world [2]. The focus of the variable speed topologies has been, in a greater extent than for the fixed speed, to maximize the aerodynamic efficiency of the turbine. This is done by adjusting the speed of the turbine to keep the tip speed ratio, defined in Section 2, as close to optimum as possible. The added controllability comes with the price of more complex systems with more components and somewhat increased installation cost. The power conversion systems also need to be efficient at variable speeds and loads if the extra gain from the variations in speed is to be utilized. With this added complexity the variable speed turbines have opened up to a large variation in both generator and power converter topologies.

There are two strong arguments for the use of full power electronic conversion systems. Firstly, the ability to control the rotational speed almost freely gives the benefits of optimal energy absorption, reduced loads, reduced noise at low wind speed as well as the possibility to use a gearless system. Secondly, the power electronics give the wind turbine the ability to be an active component in the power system [3]. This allows for control of active and reactive power flow and the ability to strengthen weak grids. This gives the wind turbine a more positive influence on the network [4, 5]. An evaluation of today's most commonly used power conversion topologies for wind power can be found in [6, 7].

The work in this paper contributes to the progress of the full variable speed wind turbines, with grid connection of the generator through a full-scale frequency converter. The electrical system needs to be able to adjust the generator voltage and frequency so that the system can deliver power to the grid. A full frequency conversion topology is proposed. The system is based on a passive diode rectifier, DC-link with a capacitor bank, an IGBT inverter, and a tap transformer. The proposed system is expected to be more robust and reliable than the conventional topologies due to lower number of active components. The proposed system can be used for any wind turbine but is in this work applied on a three-bladed vertical axis wind turbine (VAWT) with a direct driven permanent magnet generator that has been developed and built at Uppsala University [8–11]. The system is here adapted to the scaled prototype but is intended for bigger turbines where there is a real need for a step-up transformer such as the 200 kW VAWT presented in [12]. The work presented in this paper aims to evaluate the tap transformer based system topology applied on the 12 kW prototype wind turbine and its site specifics with the help of simulations and experiments.

## 2. Theory and System Overview

**Figure 1:**
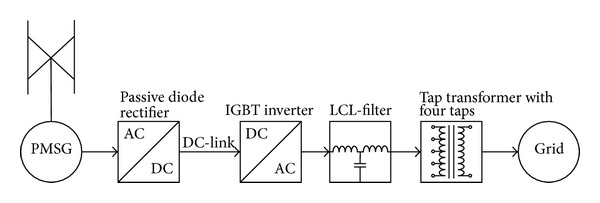
Block diagram of the system topology.

A block diagram of the system topology is shown in Figure 1. In the following text each block in Figure 1, from left to right, is described.

The vertical axis wind turbine is of the fixed pitch H-rotor type with three blades. The amount of power, *P*_*t*_, that can be extracted from the turbine in given by

(1) $$\begin{array}{c} P_{t}=\frac{1}{2}\rho ACp\left(\lambda \right)v^{3}, \end{array}$$

where *ρ* is the air density, *A* the area swept by the turbine, *Cp* the power coefficient, and *v* the wind speed. The power coefficient is a function of tip speed ratio (TSR) and represents the aerodynamic efficiency of the turbine. The tip speed ratio is defined in

(2) $$\begin{array}{c} \lambda =\frac{\omega_{t}R}{v}, \end{array}$$

where *ω*_*t*_ is the rotational speed of the turbine and *R* is the turbine radius. The *Cp* − *λ* curve for the turbine used in this paper can be found in [8].

The turbine drives a permanent magnet generator, located at the tower base, via a long shaft. The experiments in this paper are conducted in the Ångström Laboratory where a clone of the on-site generator driven by an induction motor is used. The turbine is rated to 12 kW at a wind speed of 12 m/s. The most relevant parameters for the turbine and generator are shown in Table 1. More detailed descriptions and previous work on the turbine and generator can be found in [8, 9, 13].

The voltage from a direct driven generator varies in both frequency and amplitude. This makes direct grid connection of the generator impossible. The generator is thus connected to the grid via a full frequency converter. The generator voltage is rectified using a diode rectifier without the generator neutral connected. The rectifier uses SKKD 100/12 rectifier modules rated at 1.2 kV and 100 A. The rectified current charges a capacitor bank consisting of 3 RIFA 6000_F 450 VDC capacitors that are connected so that the total capacitance is 18 mF. The mean DC-link voltage, *V*_DC_, can be derived by

(3) $$\begin{array}{c} V_{\text{DC}}=\frac{3\sqrt{2}V_{l}}{\pi }, \end{array}$$

where *V*_*l*_ is the rms value line to line voltage of the permanent magnet synchronous generator (PMSG). The DC voltage is then smoothened by a sufficient capacitor to get a mean link voltage as seen in

(4) $$\begin{array}{c} V_{\text{DC}}=\frac{2V_{l}}{\sqrt{2}}. \end{array}$$

**Figure 2:**
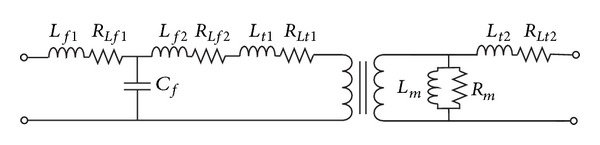
One phase equivalent circuit of the LCL-filter connected to a transformer tap.

An IGBT based voltage source inverter, controlled with sinusoidal pulse width modulation (SPWM), is used to convert the DC voltage to the desired three-phase 50 Hz AC voltage. The IGBT modules used in the inverter are of the type SEMiX252GB126HDs with Skyper 32R drivers. The control and measurements for the operation of the inverter are all done in LabVIEW using an FPGA module. The inverter output is filtered through an LCL-filter. A one phase equivalent of the LCL-filter connected to a transformer tap is shown in Figure 2. The most significant parameters are shown in Table 2. The capacitors used in the filter are of the type 24FB4460-F. The increased demand on quality of power supplied to the grid has made the LCL topology a good choice and it is suggested in several papers [14–18]. More details on the LCL-filter used in this study can be found in [19, 20]. A study of the harmonic content of a tap transformer based grid connection system is presented in [20].

The tap transformer has four different taps with the step-up ratios of 2, 3, 4, and 7, respectively. Only the three highest step-up ratios are used in this paper. The tap transformer has a star-delta configuration with the delta side connected to the grid and the taps connected in star formation to the system. The transformer values are shown in Table 3. More details about the transformer are presented in [19, 20].

In this paper the grid has been replaced with fixed resistive loads to remove the impact of the grid effects on the results. This will remove the grid harmonics and possible unbalances in the grid, as well as unwanted reactive power flow from the experiments. The resistors used are modified electric heaters that can be connected in groups to produce eight different values in the range from 13.5 *Ω* to 105.2 *Ω* per phase.

**Figure 3:**
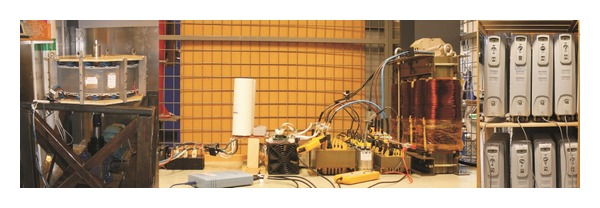
Experimental setup used to study the tap transformer topology. From the left, permanent magnet generator, inverter, LCL-filter, tap transformer, and load.

The measurements in this study were done using LabVIEW together with a NI9205 module and cRIO-9074 system. When higher sampling frequencies were needed, a PicoScope4402 was used together with the Flukei30 current clamp and Testec TT-SI9002 voltage transducer. The full experimental system can be seen in Figure 3.

## 3. Load and Site Characteristics

**Figure 4:**
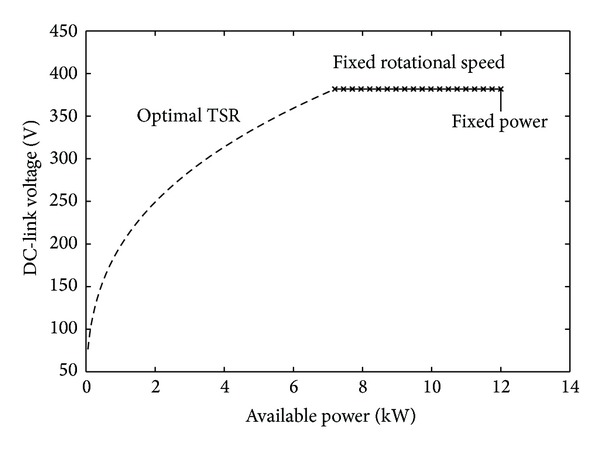
Relation between the DC-link voltage and the available power on the DC-link.

The turbine is erected at a well characterized wind site North of Uppsala, namely, the Marsta Meteorological Observatory. The Weibull parameters for the wind speed data at this site are a scale factor of 5.24 m/s and a form factor of 1.94. The Weibull probability density function is given in

(5) $$\begin{array}{c} f\left(v\right)=\frac{k}{c}{\left(\frac{v}{c}\right)}^{k-1}\exp\left[-{\left(\frac{v}{c}\right)}^{k}\right], \end{array}$$

where *k* is the shape parameter and *c* is the scale parameter. The designed control strategy for the turbine is described in [8]. The turbine is started at a wind speed of 4 m/s and run at optimal tip speed ratio from the wind speeds 4 m/s to 10 m/s. Between 10 m/s and 12 m/s the turbine is run at a fixed speed of 127 rpm. The rotational speed will still vary slightly due to voltage drops in the generator as the current is increased and due to controller limitations. The variation will be small so it can still be seen as fixed rotational speed operation. According to the designed control strategy the turbine would have been kept at fixed rotational speed until cut-out. In this study we choose to deviate from that control scheme and instead run the turbine at nominal power of 12 kW from 12 m/s until the cut-out speed of 20 m/s. This means that the DC-link voltage will drop as the wind goes from 12 m/s to 20 m/s due to the stall control that reduces the rotational speed of the turbine to keep the absorbed power constant [12]. The power curve from the control strategy together with (4) describes the relation between the DC-link voltage and the available power from the turbine, as illustrated in Figure 4.

In this paper, the efficiency of the generator and passive diode rectifier is not included in the results as we want to focus on the main part of the converter topology. The power absorbed by the turbine is assumed to be available on the DC-link. This is intended to make the system easier to compare with other systems using different generator types and designs. We define the efficiency of the system as

(6) $$\begin{array}{c} \eta =\frac{P_{\text{load}}}{P_{\text{DC}}}, \end{array}$$

where *P*_load_ is the power dissipated in the resistive load acting as the grid in the experiment and *P*_DC_ is the power drawn from the DC-link.

## 4. Simulations

**Figure 5:**
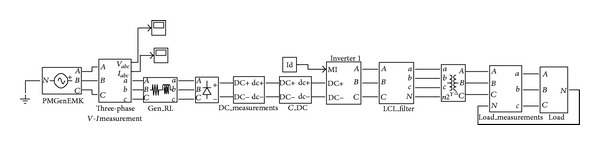
The model of the system in MATLAB/Simulink.

The system described in Section 2 was modeled in MATLAB/Simulink to assess the efficiency of the applied topology. The simulation is run so that the power drawn from the DC-link is matched with the DC voltage in accordance with Figure 4. Both the modulation of the inverter and the resistance of the load are changed to fit the load with the available power and to keep the voltage over the load at 400V Ph-Ph. The Simulink model is shown in Figure 5. The parameters and components in Section 2 are inserted into the model. The inverter is run at a switching frequency of 6 kHz and the PWM pulses are generated using a phase locked loop (PLL) and dq-controller. As the experiment uses a resistive load instead of the grid, a 50 Hz sinusoidal reference is internally generated for the PLL. Each tap of the tap transformer was modeled as a linear star-delta transformer. A transformer core saturation model is not used in the simulation; all the magnetizing and eddy current losses are represented by the magnetizing impedance.

## 5. Experimental Verification

The experiments were conducted using the generator clone in the laboratory together with the rest of the components described in Section 2. Simulations using the model shown in Section 4 were used to generate starting points for the experiments. The fixed resistors were inserted in the simulation model to collect data about modulation index and generator rotational speed for each resistance. This operational data was then used for the same fixed resistance in the experiments. Measurements to obtain the DC and AC power were performed and efficiency was calculated in accordance with (6). The same inverter control scheme, with an internally generated PLL reference, was used in the experimental setup as in the simulations.

A limiting factor for the experiments on the tap with highest step-up ratio was the current. The experimental system has a current limit of 80 A per phase. This limits the power per phase on transformer tap 1 to roughly 2.6 kW, whereas the other taps were limited by their inability to produce a sufficiently high voltage at low DC levels. A limitation for all taps was the discrete resistance values that only allowed for a few points in the operational range.

## 6. Results and Discussion

**Figure 6:**
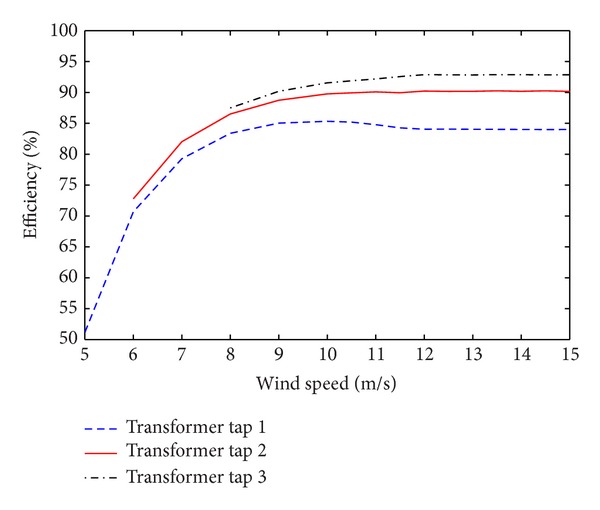
Simulated system efficiency as a function of wind speed.

The results from the simulations are shown in Figure 6 where the different taps are shown in their full operating range. The efficiency is lowest at low wind speeds and increases with increasing wind speeds. It levels out when the fixed power region of operation is reached. This is expected, as all major losses are constant and we only have a slight increase in current as the DC-link voltage decreases. Furthermore, we see in Figure 6 that the efficiency at nominal power is increased by roughly 6% by going from transformer tap 1 to tap 2 and by approximately 8% by changing from tap 1 to tap 3 which is a significant increase.

**Figure 7:**
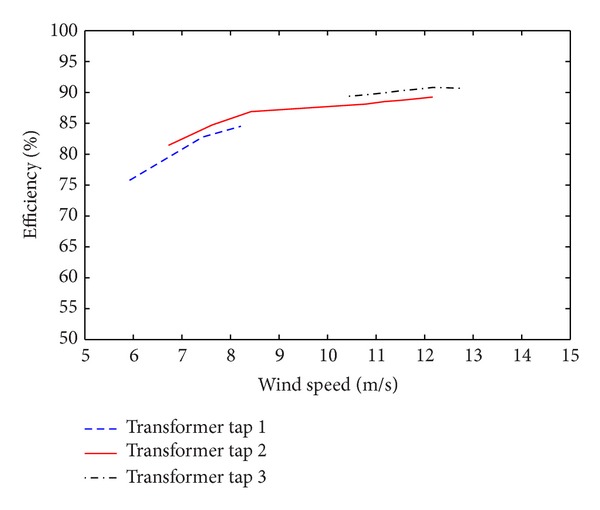
Experimental system efficiency as a function of wind speed.

The results from the experiments are shown in Figure 7. The range of the experimental data is limited by the fixed values of the resistive load. However, all operating points included in Case 2 below are included. Here we see the same trends in efficiency as in the simulated results but with a deviation of roughly 2%; see Figure 9. The deviation can be caused by a number of different things such as measurement errors, the low order frequency model for the transformer and filter coils. Some of the differences can be explained by a possibly unbalanced transformer, saturation effects, and leakage in filter coils and transformer, not included in the simulation. However, we still see a clear increase in efficiency as we change tap and the same trends in both the experiments and simulations.

### 6.1. System Losses Distribution

**Figure 8:**
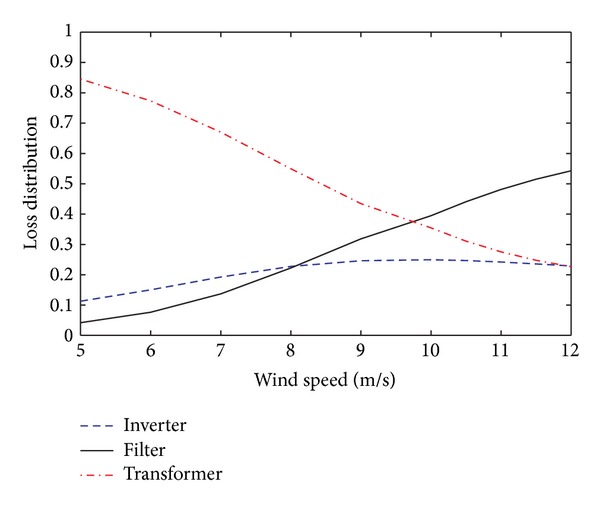
Loss distribution of the simulated losses in the system when run at tap 1 for all wind speeds as a function of wind speed for the main system blocks.

The system losses vary strongly with the wind speed. At low wind speeds the transformer losses are dominant; see Figure 8. This is to be expected as the magnetization losses of the transformer core are fairly constant, implying that at low wind speeds and low power the transformer losses will dominate. The choice of transformer thereby becomes a trade-off between the desired transformer power rating and the system efficiency at low wind speed. In this study a 16 kVA transformer is used as the experimental setup is designed for a higher power than the turbine power rating. If we had used a transformer rated close to nominal power we would have increased the overall efficiency of the system especially at low wind speeds. At higher wind speeds the transformer losses play a smaller part. Instead, the increase in current and the pulse width modulation give increased losses in the inverter and filter. Hence, a trade-off is made between filter values and inverter switching frequency, as a higher switching frequency generally gives higher inverter losses and lower filter losses. The experimental system in this study is designed to work and give good results over a wide range of switching frequencies. If it had been optimized for operation at 6 kHz, losses could be reduced further. This implies that the efficiency shown in Figures 6 and 7 can be further improved by fine tuning the system but the overall trend would still be the same.

### 6.2. Case Study

In this case study we examine different tap selections and compare different alternatives for how to run the turbine. To get a proper understanding of the average power output of the system, four cases are chosen. For each case, the Weibull site parameters described in Section 3 are combined with the simulated efficiency and control strategy to get the average power output. To give a more general overview, the shape factor in the Weibull distribution is varied in each case. It would be unwise to only consider the rated power as it, on this particular site, only occurs a small fraction of the time.

*Case  1*. Here we assume a standard transformer that can deliver power to the grid at all wind speeds. This is represented by running the system only using tap 1 on the tap transformer for the full operational range of the turbine.

*Case  2*. The system is run using all available taps and the tap change is done as soon as a tap with a higher efficiency is available, with some hysteresis implemented. In winds from 4 m/s until 7 m/s tap 1 is used, from 7 m/s until 9 m/s tap 2 is used, and for the rest of the wind speeds tap 3 is used.

*Case  3*. The low wind speeds are ignored and only taps 2 and 3 are used. In this mode of operation the wind turbine does not start to deliver power to the grid before a wind speed of 6 m/s and the tap change from tap 2 to tap 3 is done at 9 m/s.

*Case  4*. In this scenario tap 2 is excluded and the turbine is run with taps 1 and 3. In winds from 4 m/s until 9 m/s tap 1 is used and for the rest of the wind speeds tap 3 is used.

**Figure 9:**
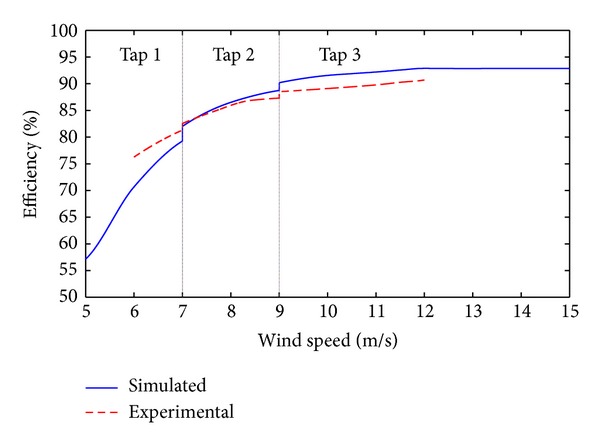
System efficiency for Case 2.

The increase of power output for the four cases relative to the base case, Case 1, is presented in Table 4. The results from the case study show an increase in overall power by roughly 5% for Case 2 at our site with *c* = 5.24 and slightly above 3% for Case 4 compared to the base case. This is a significant increase in production. However, these results are very site specific and for a wind site with higher mean wind speeds a higher utilization of the high taps is achieved, giving an even more significant impact on the overall power production as seen in Table 4. Case 3 only becomes an option at sites with high mean wind speeds. The system efficiency for the proposed operation in Case 2 is illustrated in Figure 9 for both simulations and experimental results. A jump in efficiency as we change between taps can be seen.

**Figure 10:**
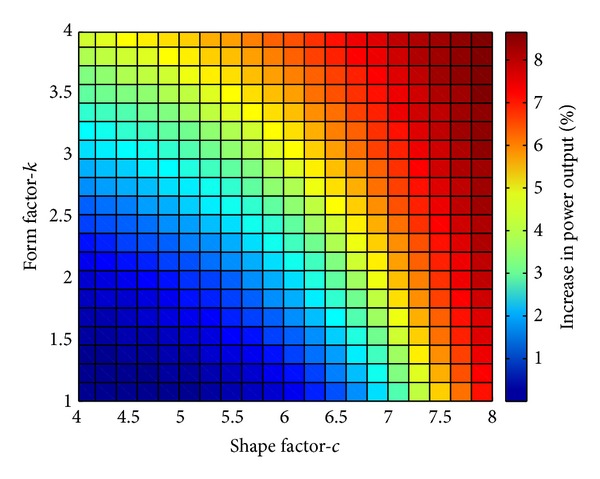
Sweep of relative power output for changing form factor and shape factor in the Weibull distribution for Case 2 relative to Case 1.

A sweep in both form factor and shape factor together with the operation described in Case 2 is shown in Figure 10. The results of the sweep confirm that the overall system efficiency is higher at high wind speed sites. Further, these results show the importance of considering the site when designing the electrical system.

## 7. Conclusions

A novel electrical system configuration for variable speed wind turbines has been presented and demonstrated. The system has a high efficiency at nominal power and the system efficiency at nominal power is increased by going up in tap. The tap transformer topology gives an increase in overall power production in comparison with a standard transformer. It is concluded that the magnetizing losses of the transformer dominate at low wind speeds. Further, the wind site needs to be considered already at the design stage in order to do a proper system evaluation.

## Figures and Tables

**Table 1:** Turbine and generator parameters.

Optimal tip speed ratio	4
Rated power (kW)	12
Nominal phase voltage (V)	161
Nominal electrical frequency (Hz)	33.9
Per phase generator resistance (Ω)	0.151
Per phase generator inductance (mH)	2.6

**Table 2:** LCL-filter and tap transformer values.

Inverter side coil *L*_*f*1_ (*μ*H)	400
Inverter side coil resistance *R*_*Lf*1_ (mΩ)	23
Transformer side coil *L*_*f*2_ (*μ*H)	150
Transformer side coil resistance *R*_*Lf*2_ (mΩ)	11
Filter capacitance *C*_*f*_ (*μ*F)	120
Magnetization resistance *R*_*m*_ (kΩ)	1.8
Magnetization inductance *L*_*m*_ (H)	1.6

**Table 3:** Tap transformer parameters.

Tap	1	2	3
Step-up ratio	7	4	3
Input voltage Ph-Ph rms (V)	57	100	133
Output voltage Ph-Ph rms (V)	400	400	400
Power rating (kVA)	16	16	16
Filter side coil *L*_*t*1_ (*μ*H)	28	51	88
Filter side coil resistance *R*_*Lt*1_ (mΩ)	9	16	21
Grid side coil *L*_*t*2_ (*μ*H)	64	64	64
Grid side coil resistance *R*_*Lt*2_ (mΩ)	121	121	121

**Table 4:** Power output in percentage for the different cases relative to the base case, Case  1, for different scale factors, *c*.

Case	1	2	3	4
*c* = 4	100%	102.6%	47.2%	101.0%
*c* = 5.24	100%	104.5%	76.3%	103.3%
*c* = 6	100%	105.7%	86.6%	104.6%
*c* = 7	100%	106.9%	94.7%	106.0%
*c* = 8	100%	107.7%	99.3%	107.0%

Taps	1	1, 2, 3	2, 3	1, 3
